# Treatment Experiences with Norwegian Health Care among Immigrant Men Living with Co-Occurring Substance Use- and Mental Health Disorders

**DOI:** 10.1177/1178221820970929

**Published:** 2020-11-24

**Authors:** Prabhjot Kour, Lars Lien, Bernadette Kumar, Stian Biong, Henning Pettersen

**Affiliations:** 1 Norwegian National Advisory Unit on Concurrent Substance Abuse and Mental Health Disorders (NK-ROP), Innlandet Hospital Trust and University of South-Eastern Norway, Norway; 2 Norwegian National Advisory Unit on Concurrent Substance Abuse and Mental Health Disorders (NK-ROP) Innlandet Hospital Trust; and Faculty of Health and Social Sciences, Inland Norway University of Applied Sciences, Norway; 3 Norwegian Institute of Public Health, Norway; 4 University of South-Eastern Norway, Kongsberg, Norway

**Keywords:** Co-occurring disorders, mental health disorder, substance use disorder, immigrants, qualitative methods, treatment experiences, lived experiences

## Abstract

Immigrants are considered at risk of psychological distress and therefore involvement in substance abuse, due to a variety of pre- and post-migration factors. Further, there is lower treatment engagement, a higher dropout rate, and less frequent hospitalizations among this group compared to the general population. There are few studies on the subjective understanding of co-occurring substance use disorder (SUD) and mental health disorder (MHD) among immigrants in Norway. This qualitative study aims to explore the treatment experiences of immigrant men living with co-occurring SUD and MHD. Within a collaborative approach, individual interviews were conducted with 10 men of immigrant background, living with co-occurring SUD and MHD, who had treatment experiences from the Norwegian mental health and addiction services. Data were analyzed using a systematic text condensation. The analysis yielded 6 categories where participants described their treatment experiences in mental health and addiction services in Norway as: lack of connection, lack of individually tailored treatment, stigma and discrimination preventing access to treatment, health professionals with multi-cultural competence, care during and after treatment, and raising awareness and reducing stigma. A significant finding was the mention by participants of the value of being seen and treated as a “person” rather than their diagnosis, which may increase treatment engagement. They further mentioned aftercare as an important factor to prevent relapse. This study provides an enhanced understanding of how immigrant men living with co-occurring SUD and MHD experienced being treated in Norwegian healthcare settings. These experiences may add to the knowledge required to improve treatment engagement.

## Introduction

Over the past 2 decades Norway has become a multicultural and ethnically diverse society due to a substantial increase in migration.^1^ Immigrants account for 18.2% of Norway’s population, and 10.8% are from middle- and lower-income countries.^2^ Previous studies show that immigrants may be at risk for developing mental health problems due to a variety of pre- and post-migration factors.^1,3^ Mental health problems and substance use often present with a high degree of co-occurrence^4-6^ with poor life quality^7^ and those diagnosed with co-occurring substance use disorder (SUD) and mental health disorder (MHD) are often referred to as “complex” and “difficult to help” due to lack of tailored services.^8^ SUD is understood as a term that includes harmful use of, and dependency on, drugs and alcohol^9^ and MHD as range of problems characterized by some combination of abnormal thought, emotions, behaviors and relationships, for example, depression, schizophrenia, intellectual disabilities, post-traumatic stress disorder etc.^10^ Further, co-occurring disorder is understood as the co-existing of SUD and any combination of MHD in an individual with a strong impact on everyday life.

Further, research has documented that immigrant groups with SUD and MHD are at a high risk of neglect even in developed healthcare systems; possible reasons include lack of existing healthcare policies for these groups and insufficient funding to target specific areas of immigrants’ mental health care.^11^ A recent Swedish cohort study reported increased rates of SUDs among immigrants who migrated at an early age or had lived for a long time in the host country^12^; they were further disadvantaged by poor access to healthcare.^13,14^ In addition, immigrants who have connections to a closely-knit drug scene or group may be hampered from seeking mental health treatment. This could be due to the feeling of social solidarity that they want to display within the group. Further, these closely-knit scenes may provide a community for immigrants who experience a low degree of inclusion elsewhere.^15^ Moreover, it has also been documented that immigrants have lower rates of utilization of specialist mental health care services in Norway,^16^ compared to the general population. This has also been documented among immigrants in Sweden,^17^ and among non-Western immigrants in the Netherlands.^18^

There is strong evidence that completion of SUD treatment is one of the most consistent factors associated with favorable treatment outcome.^19^ Persons living with SUD and MHD are often difficult to engage in treatment, leading to frequent relapses and rehospitalizations.^20^ Lower treatment engagement has also been documented among immigrants,^21-23^ resulting in challenges in providing them with targeted help.^15^ Engagement is understood as the process of establishing a mutually collaborative, trusting, and respectful helping relationship.^24^ Integration of principles of person-centered healthcare (PCH) in mental health and addiction services have been shown to enhance the engagement process and lead to improved outcomes.^20^ Person-centered healthcare focuses on the unique goals and life circumstances of an individual in MHD and SUD treatment models^20^ by not only managing and overcoming the health conditions, but also rebuilding the lives of persons living with SUD and MHD.^25^

Further, PCH involves valuing the individual as a “person” with objective, absolute and intrinsic worth,^26^ along with the person’s life history and relationships, both in illness and health.^27^ We conceptualize “person” as defined by Cassell, “as an embodied, purposeful, thinking, feeling, emotional, reflective, relational, human individual always in action, responsive to meaning and whose life in all spheres points both outward and inward. Virtually all of a person’s actions – volitional, habitual, instinctual or automatic – are based on meanings”.^28^ Considering the fact that “meaning” and “personhood” are mutually constituted, understanding about persons involves understanding about values and social phenomena.^29^ This may imply that treatment engagement will be improved by the development, coordination and provision of healthcare services that respect the uniqueness of individuals by focusing on their values, beliefs, desires and wishes, regardless of their age, gender, social status, faith, financial situation, ethnicity, and cultural background.^30^ We believe that this holds true for immigrants living with co-occurring MHD and SUD, who often feel discriminated against, stigmatized, left alone, and lack a sense of belonging.^31^ PCH has emerged as a cornerstone of effective SUD treatment^32,33^ and has been highlighted in the Norwegian national guidelines for SUD treatment.^34^ In addition, we believe that culturally tailored healthcare services are a part of person-centered “cultures”, which may lead to higher treatment engagement.

Various studies have shown that immigrants are less likely to use the available mental health and addiction services,^16,35,36^ which may be due to lower treatment engagement and unsatisfied treatment needs. There have been a few quantitative studies, but no qualitative research, that has studied treatment experiences from the perspectives of immigrants. Thus, there is a knowledge gap concerning immigrants’ subjective experiences of MHD and SUD treatment.

This descriptive and exploratory study aims to examine the treatment experiences of immigrant men living with co-occurring MHD and SUD in Norwegian mental health and addiction services.

## Methods

### Collaborative study design

The study follows a qualitative and exploratory design with a collaborative approach. Traditional research into mental health and substance use disorder treatment is considered by an increasing number of service users as disempowering, and poorly reflective of their priorities.^37,38^ To remedy this, the present study has adopted a collaborative research approach. Collaborative research brings a different perspective to the research process, which is highly relevant to clinical practice and helps to improve the evidence base used to inform how services are provided.^39^ Thus, a competency group of 3 persons was established to work with the research team in all stages of the study. Two members were previous users with lived experience of having co-occurring MHD and SUD, and one was a relative of one of the users. All 3 members were of immigrant background and had an understanding of both their original local context as well as the Norwegian context.

### Recruitment

A criterion-based, purposive sampling method^40^ followed by snowballing was employed to recruit the participants from 2 cities in Norway. The inclusion criteria were persons with immigrant background, from low- and middle-income countries, having experienced living with co-occurring SUD and MHD and treatment in Norwegian healthcare. A diverse sample was included in order to obtain information-rich data for the study. “Immigrants” in this project are understood as persons who were born or whose parents were born in low- and middle-income countries. In addition, we include as immigrants persons born abroad or in Norway of 2 foreign-born parents and 4 foreign-born grandparents in our study.^2^ We would also like to point out that “immigrants” are not a homogenous group; they differ in various ways, including culture, ethnicity, reason for migration, historical migration patterns, etc.

Recruitment began by phoning and sending emails to leaders of various rehabilitation and treatment centers in these 2 cities. The facilities chosen were those with access to potential participants with the inclusion criteria. Detailed information about the research project was given to these leaders. The recruitment of participants meeting the inclusion criteria was more challenging than expected. We do not know exactly how many persons were asked by team leaders to participate, but most of them refused to take part in the study. A few reasons mentioned were, person’s unwillingness to talk about their lived experiences, lack of trust in system, including any research project, fear of being detected in their small immigrant communities in Norway and fear of stigma attached to SUDs and MHDs and seeking it’s treatment. Further, through these leaders only one participant was recruited. This participant was contacted by telephone by the first author and given information about the study. Subsequently, by snowballing, this participant helped to recruit 3 more participants with whom he had contact. The competency group played a key role in recruiting further participants. Six participants were recruited with the help of the competency group, whose members had contacts in their local community. Potential participants were able to show interest by contacting the first author by telephone or SMS.

### Study participants

The study included 10 participants who met the inclusion criteria (Appendix A, Table 1). All the participants were males, aged from 25 to 53 years. All of them had been diagnosed with co-occurring SUD and MHD and had experience of treatment in the Norwegian mental health and addiction services. All participants were polysubstance users and the most common substances used were alcohol, heroin, and cannabis. Five participants reported not using substances at the time of the interview. The participants also stated having experienced MHDs, most commonly anxiety, affective disorder, personality disorder and post-traumatic stress disorder. Eight of 10 participants reported having experienced suicidal thoughts and suicide attempts. The objective diagnosis was not considered, but rather how the participants understood their own mental health condition. Five participants were from the Middle East, while others were from South Asia and East and West Africa. Two participants were born in Norway, and most others migrated at a very early age, while 2 had arrived at age 21 and 24. All the participants started using substances at an early age: 8 after arriving in Norway, while the 2 who migrated at age 21 and 24 started at a young age in their countries of origin. All the participants had dropped out of treatment in Norway at least once and most had dropped out several times. All participants had the treatment seeking experience in one or the other mental health or/and addiction services, such as generic specialist service units, special units that collaborate closely with primary level services, residential addiction treatment based on AA- or NA-principles and detoxification units. Further, participants have both in-patient and out-patient treatment experience, where the length of treatment program ranged from 9 to 12 months, and some of the participants were still continuing the treatment.

### Data collection

Data were collected through in-depth, semi-structured individual interviews^41^ between June 2018 and March 2019. The data were considered be sufficient to fulfill the aim of the study after the tenth interview. This was done using the concept of “information power”, which implies that the more relevant information the sample has, the smaller the number of participants needed.^42^ This could be justified in our study as the aim of the study was narrow, and concerns a specific experience among a population that hold specific characteristics and which are “hard-to-get” group, which would in itself limit the number of eligible participants (diverse immigrant background, living with co-occurring SUDs and MHDs, with having treatment experience in Norwegian mental health and addiction services). Further, we believe that participants hold the experiences that have previously not been described, has also enhanced the information power. Also, there was strong and clear communication between the first author and the participants, partly due to being introduced by the members of competency group and partly because the first author has previous experience with qualitative interviews. Moreover, the after the tenth interview, assessment was made by all the authors that these data made it possible to answer the aim of the study. All the interviews were audio taped and lasted from 40 to 90 minutes. Nine of the 10 interviews were conducted in Norwegian by the first author and an interpreter, while one interview was carried out in Punjabi and English by the first author only. All the interviews were transcribed in English. An interview guide, consisting of open-ended questions about what it means to be treated in Norwegian healthcare settings when living with co-occurring disorders as a person of immigrant background, was created and agreed upon by all authors and the competency group. The main questions included were (all these questions had the follow up and probing questions): Can you please describe your experiences with treatment within Norwegian mental health and addiction services as a person with immigrant background? Can you please tell us why according to you persons with immigrant background do not take the complete treatment? Can you please describe your experiences with treatment when it comes to your needs as a person with immigrant background? What kind of treatment do you wish for as a person with immigrant background in Norway?

### Data analysis

The interview conducted in English and Punjabi was transcribed by the first author and the remaining 9 conducted in Norwegian were transcribed by the interpreter. These interview transcripts were analyzed using systematic text condensation (STC),^43^ which is a descriptive and explorative method which aims at thematic cross-case analysis, and which maintains methodological rigor and enables feasibility, intersubjectivity, and reflexivity. STC is a stepwise procedure that includes the identification of recurring initial codes and themes relevant to the aim of the study. Step one involves the formulation of a total impression gained by reading all the transcripts, leading to initial themes. In step two, after systematically reviewing the transcripts, meaning units were identified and sorted into code groups. The third step involved the formation of subgroups from code groups with meaning units. The next step was to form artificial quotations by the reduction of meaning units under each subgroup. In the final step, analytic text and descriptions were developed from artificial quotations. The analytic text was reconceptualized by returning to the complete transcripts and reflecting on whether each illustrative quotation still reflected the original content. This was done in order to validate the analytic texts. Lastly, the analytic texts were supported by quotes, which are presented in the “Results” section. In each step, all the co-authors were consulted and discussions took place. In the final step, the competency group was consulted to provide an understanding of the results within the local context they represented.

### Ethical aspects

The study was ethically approved by the Norwegian Centre for Research Data (Project No. 59707). The research procedure was designed and followed in accordance with the Declaration of Helsinki. The participants agreed to take part in the study voluntarily and signed the informed consent, which ensured their confidentiality and anonymity. They received an information letter and an oral explanation about the project prior to the interviews. The members of the competency group and the interpreter signed a confidentiality declaration. The participants were given the contact details of the first author in case they had any concerns or questions after the interviews.

## Results

The analysis yielded 6 categories where participants described their treatment experiences in mental health and addiction services in Norway as: lack of connection, lack of individually tailored treatment, stigma and discrimination preventing access to treatment, health professionals with multi-cultural competence, care during and after treatment, and raising awareness and reducing stigma. A significant finding was the mention by participants of the value of being seen and treated as a “person” rather than their diagnosis, which may increase treatment engagement. They further mentioned aftercare as an important factor to prevent relapse.

### Lack of connection

A majority of participants experienced a lack of connection between themselves and health professionals (HPs) while they were in treatment, such as a lack of interest from HPs. The feeling of lack of connection was interpreted as not being listened to and having unsatisfactory communication. They further mentioned that the lack of connection was stronger when the HP only talked about their diagnosis and said nothing about contexts that were important for them, like their process of migration, living as an immigrant in Norway, reasons why they started using substances and discrimination in treatment settings. They experienced not being seen as a person in treatment settings, leading to a more pronounced feeling of lack of connection, which discouraged them from continuing the treatment.

*They did not understand my needs. It was only written in papers, about me, that my mother is divorced and about events of my life but there were actually no conversations about it with me, nothing about what it’s like to be an immigrant. . . (P-6)*

The participants also stated that there was no aftercare once they were out of the treatment centers. They had no contact with service providers after the standard treatment was over. They had to follow a schedule while they were in treatment and they felt lost once they had no schedule, leading to a higher chance of relapse.

*We didn’t have any aftercare. When people are done, what happens to them afterwards? It’s called aftercare, in the medical terminology. And that’s the part we are bad at. The time when you are inside, you know that 12 o’clock you go and eat, 6 o’clock the food comes on the table, 9 o’clock is dinner time. You have a routine. But when you come out, nobody tells you to go and eat at 12 o’clock, do they? And if you’re hungry, or thirsty, all that, it can have almost the same effect as the drugs, you see?. . .But when you wake up in the morning, look around, see the same apartment, the same place, the same things, you know? They can’t take it. And then you go and you take drugs. . .(P-8)*

Participants also described how HPs did not have any understanding of their cultural context, which meant that their needs were not met in treatment, further leading to a lack of connection between them and the HP.

*If you talk about immigrant background, there is actually a lack of cultural identification topics in every treatment I have been to in Norway, both in psychiatric and drug abuse treatment. They do not have this in their protocol. I will give you an example, I know so many people from Iceland who have addiction problems, it is a big issue with them, they are alcoholics. I have many friends who are from Iceland and we talk about this topic many times among ourselves and they also say they want more culturally specific treatment, that we do not have in Norway. (P-3)*

### Lack of individually tailored treatment

The participants described experiencing a conventional and standard approach to treatment with no cultural sensitivity. There were no new strategies brought up in the treatment protocol that could satisfy their needs of being culturally different from the majority. They mentioned having few daily activities and a monotonous routine while in treatment, which discouraged continuity of treatment. Further, they mentioned conventional group therapy where the participants felt mismatched in the group. This led to discomfort and lack of satisfaction in the group therapy. Some participants also mentioned that even though they had the same sickness and diagnosis as Norwegians, their needs were different. They further added that even though group therapy was important, individual therapy was equally important, because of their different needs and levels of understanding, and they had not experienced that as part of the treatment.

*I felt bad, I felt like shit. The others were old, in their 40s and had been shooting needles for years. I was only 20 so I felt very small, I felt dirty, small, low. It wasn’t a good place to be. You just sat there and ate and watched TV. I thought to myself “Is this the place to be for treatment?”(P-1)*

Furthermore, participants narrated their experiences of not being understood specifically in relation to their cultural beliefs, values and language. Only being prescribed the standard anti-depressant and sleeping pills when they asked for help, without the HP understanding their social and cultural context led to their discontinuing the treatment. Some participants expressed worries as they felt vulnerable being an addict and might be very likely to become dependent on sleeping pills.

### Stigma and discrimination preventing access to treatment

Living with SUD and MHD, all the participants described experiencing stigma and discrimination in one form or another. They were aware of the fact that using substances and having MHD was associated with stigma and discrimination, which had restrained them from accessing help and adhering to treatment. If they sought help, they felt they would become even more stigmatized in their small communities.

*I see so many Norwegians in the treatment, of course they are in the majority here but very few immigrants. They feel that they will be stigmatized if they take such treatments, they want to be identified beyond their skin color, beyond their social and cultural background. That is one of the main reasons they don’t take treatment. . .. That makes me feel inferior, especially with such closed and cold behavior. That’s the biggest stigma and this stigma is also incorporated in the Norwegian health care system. (P-6)*

Many participants revealed a cultural stigma in acknowledging the problems of SUD and MHD, and this prevented them from seeking treatment and help. They described that seeking psychiatric help was associated with a stigma in their culture where they were labeled “mad”. They also stated that they had to drop out of treatment because of family pressure and the stigma that the family faced, which led to relapses and continued use of substances.

In addition, participants recounted experiences of discrimination that they faced while they were in the treatment centers, due to their immigrant background, which also prevented them from getting the help they needed in treatment. They mentioned incidents of being looked down upon by the HPs, which led to them dropping out of treatment and further relapses.

*So I went to the meeting there and there was a psychiatrist there and she was going to talk to me and I shared a bit about my life and such, about how I had been treated here in terms of racism. I was so vulnerable and then she only asked me “Are you violent?”, and I thought “What. . . are you going to start judging me?”, that’s what I thought at least. I said I can’t work with you. You are already judging me when I am so vulnerable and then asking me if I’m violent. I’ve had enough of that violent thing. Every time I have been seen in the streets they asked if I was violent. What does that have to do with anything? I also want to live like a person in this country, I don’t want to have these labels on me all the time. That’s what hurts, right? (P-8)*

### Health professionals with multi-cultural competence

Several participants experienced that having health professionals who had expertise in understanding different cultural backgrounds was helpful in completing treatment. They further mentioned that HPs who recognized the special needs of immigrants led to a higher level of satisfaction with treatment. In addition, participants stated that it could lead to better treatment outcomes if HPs learned more about different cultures via seminars and shared results of different studies and experiences from treatment centers in Norway and abroad that have successful rates in treating persons with different cultural backgrounds. Moreover, participants reported receiving the most help from HPs who had an understanding of both foreign and Norwegian culture. Experiences of having being understood and accepted as who they were without judgments in the treatment centers were facilitators for their treatment.

*He (HP with immigrant background) is quite well-known in the psychologist circles, and he works as chief psychologist, here, which is the oldest place offering treatment in Norway. It was really great. I just met him and I felt it was positive. .. He had experience with people who had seen war, and that helped, yes. (P-10)*

Some participants also stated that having an HP with an immigrant background was a motivating experience, as it became easier to connect with them. They experienced a sense of being better understood by HPs with a similar background, which helped in building trust. Further, a few participants mentioned that having an HP with experience of war traumas was beneficial for them, as they could open up about their own trauma experiences. This provided participants with motivation to complete treatment and a sense of belonging.

*Member of staff or doctor with a similar background. . . That would be perfect. . .definitely because there was one health professional from Chile at the treatment center where I was admitted to. I could identify with him, much better than with other health workers. . . I opened up a little bit with health workers from different cultural backgrounds, on the sole basis of identification, that I could identify with them. (P-2)*

### Care during and after treatment

Experiences of being valued as a person in treatment centers, and not being looked down upon based on their diagnosis and their immigrant background, were regarded as meaningful. One participant described the feeling of being well attended to by one of the HPs and not seen as an “addict” who does not look like most people in that treatment center, and has different cultural background. The participant experienced the positive feeling of being welcomed and thus completed treatment in the same center twice. A few participants said that conversations about their past related to migration and settling in Norway and understanding their needs in relation to their past in the treatment process had a positive impact on them. Another participant also mentioned that the use of prayers in treatment gave him the experience that life is meaningful and worth living, which helped him to adhere to treatment.

*Well, if someone would sit there alone, or looked a bit depressed, he (HP) would try and involve the person a bit more. He would say “Come here”, and he would walk into their rooms and bring us out and things like that. He kind of involved us, he tried to pull us out of those thoughts, feeling included was a good thing. (P-3)*

For a few participants, the experience of being treated with love and respect regardless of their cultural background was encouraging and this created an open and healthy relationship between them and the HP. Moreover, positive attitudes of HPs with expressions of gratitude toward the participants, like shaking hands and hugging, contributed to a higher level of satisfaction with treatment. This further led to the building of trust between the participants and HPs.

*Luckily the people (HPs) that worked there said “Try telling us a little, you have the same rights as us here in Norway.” And I thought “Oh, do I? Can I also take a little space here?”, And I started thinking, oh maybe I can. Maybe the woods outside are also for me, and not only for Norwegians. Maybe it’s not just Norwegian nature for Norwegians. Maybe I can actually enjoy the green leaves as well. That’s how I began, step by step. (P-8)*

Most of the participants mentioned the importance of aftercare. They described being lost after they left the treatment centers, they had no schedule and no one to look after them, which ultimately led to frustration and relapses. Further, they mentioned that aftercare was as important as in-treatment care. This was because they did not spend a long time in treatment or detoxification centers and were thus on their own most of the time and were more likely to have relapses when they were not followed up, especially because they had different cultural background and lack the feeling of social inclusion elsewhere. A few participants mentioned that aftercare from HPs was crucial in their treatment process, as they often remained hidden and could not ask for help in their small communities due to fear of stigmatization.

### Raising awareness and reducing stigma

Several participants stated that it would have been easier for them to access treatment if there was less stigma attached to SUDs and MHDs among acquaintances and in their communities. One participant mentioned that it would be his dream treatment if he could be seen and treated beyond the wall of the stigma in healthcare and his community. Others mentioned that it would be better not to have stigmatizing names of treatment centers, like ‘acute addiction ward’, but to have nicer names, as they felt that such traditional names were associated with stigma within their community and prevented their access to the services needed for emergency treatment.

*Take away some of that shame. . . To talk more about it in the media, maybe? To remove that taboo that is there. We have to be a bit realistic and admit that a drug/alcohol problem is seen as a taboo still, and a moral problem, not a sickness, actually. . . That barrier can also be removed just by seeing it as a sickness, you know what I mean. I think that many people, me included, have way too much prejudice when it comes to drugs and alcohol. (P-1)*

Obtaining information and awareness about the consequences, diagnosis and treatment strategies from the HP was described as a positive experience by a few participants. They described how having insight into their diagnosis and the harmful effects of using substances motivated them to adhere to and complete treatment. Further, some participants mentioned that raising awareness about the harmful effects of substances and available treatment options, and reducing stigma via the media, would allow their co-users to access treatment. In addition, they stated that raising early awareness in primary and secondary schools was important, as most of them had started using substances at that time.

*and at the same time, a treatment where you get information. Information about your sickness, what are the drugs doing to you, how are they making you react, why do you, despite being sick the day before, go out and buy the same stuff and become as sick as before, again. What is that insanity? Those places where they can give you information and take care of you, at the same time as you yourself have to create a network. These are the places I go for. . ..(P-3)*

Additionally, having a supportive network of family, peers and role models was mentioned as particularly helpful, both during and after treatment. A few participants mentioned that this supportive network was key to giving them hope, motivation and a positive approach toward accessing and completing treatment. This further gave them a feeling of inclusion which was a driving force to get out of their “dark side of life” and change their self.

*Encouragement. Because I had psychologists that encouraged me and I had those who think negatively themselves and it was contagious. So, it is important, very important for the patients, to understand that it is possible to get well. It is the most important thing. I would say that it is to try to build trust in the patient. (P-9)*

## Discussion

In this study, we explored the treatment experiences of men of immigrant background living with co-occurring SUD and MHD in Norwegian mental health and addiction services. Six main categories of experiences were revealed, which we classified into 2 major insights. First, negative experiences that acted as barriers and reduced treatment engagement. These negative experiences posed significant challenges for the participants prevented them from seeking treatment and encouraged drop-out. Emphasis was placed on the connection between them and the HPs, individually tailored treatment and stigma and discrimination. Secondly, positive experiences that functioned as facilitators and enhanced treatment engagement. HPs with multicultural competence, being cared for well during and after treatment, along with strategies to reduce stigma and raise awareness for treatment completion were supportive experiences for the participants’ well-being. Further, a significant finding of being treated as a “person” in treatment settings, not as a disease or diagnosis, was reflected in both types of experiences. Being regarded as a “person” was experienced as positive and valued, while not being seen as a “person” was perceived as negative and led to lower treatment engagement.

Participants’ experiences of not being treated as a “person” in the treatment setting resulted in lower treatment engagement. They described the notion of “person” as being valued and respected for who they were, which was dependent on their immigrant background and their life history. They had been through a series of disruptive events (immigration, living with SUD and MHD) which had shaped their coping and negotiating of their sense of self.^31^ They experienced a lack of discussions around events such as their migration process, initiation of substance use and coping with stigma and discrimination in community and treatment settings, which were vital for them. They felt of little worth when conversations only focused on their diagnosis and symptoms, which made it impossible to feel like a “person” while they were in treatment. This could be understood in terms of Cassell’s definition of “person”, which involves understanding an individual as a person in medicine, based on the meanings of the person’s actions^28^ through an understanding of values and beliefs of that person in a particular social context.^29^

Further, participants’ experiences of lack of connection with HPs in view of negative past encounters with lack of interest and little attention in treatment sessions resulted in lower treatment engagement. Other studies have found that many immigrants are reluctant to seek treatment for mental health and substance use problems, which results in poor health outcomes with longer duration of untreated problems.^44,45^ This could be attributed to difficulty in developing trust in mental health and addiction services due to unfamiliarity with how these services work^31,46^ and previous negative experiences with treatment.^35^ Moreover, participants revealed that the situation worsened when they had to wait longer to start treatment, leading to more substance use and relapses, which is reported by Pinedo et al.^36^ as a logistical barrier to treatment.

Another barrier that overwhelmingly shaped participants’ decisions not to enter treatment or to be less engaged in treatment was the lack of culturally competent services tailored to their specific needs in relation to cultural beliefs, values and language, which is in line with previous studies.^36,45^ Misunderstandings arise regarding patients’ acceptable and typical behavior when HPs lack cultural competence.^46^ This often leads to a lack of discussions on important social contexts, such as immigration and discrimination within treatment settings, resulting in low adherence rates,^36^ which concurs with the participants’ experiences. Furthermore, the treatment program of 12-step support group therapy did not function well with the participants as they felt the groups were mismatched with regard to age, language and duration of using substances. They further mentioned that they had difficulty expressing emotions and sharing private information within such groups and hence showed lower treatment engagement, as in a study on Asian immigrants in the US.^45^ Connected to this is the lack of individually tailored services for immigrants, which reduces their engagement to treatment. This could be due to the manual-based and standard trend of knowledge-based practice that is followed in mental health and addiction services, which may lack individualization and cultural sensitivity within treatment.

Further, experiences of stigma prevented participants from accessing treatment when living in small communities in Norway. This is in line with previous studies stating that stigma regarding seeking treatment for SUDs and MHDs was a significant barrier to enroll in treatment programs. These disorders among immigrants are often viewed as a sign of weakness, shame or a lack of willpower, which often results in ambivalence about seeking timely help; either the person delays or does not seek treatment at all.^45,47,48^ Further, in recent studies on immigrants, fear of being negatively perceived within their community, especially family resistance, discouraged them from seeking treatment even though they were willing to get help,^31,36^ which concurs with the participants’ experiences in our study. In addition to stigma, our participants mentioned experiences of discrimination and unfair treatment from HPs which lowered their treatment engagement. This further resulted in a higher risk of relapses and severe mental health disorders.^47,49^ These experiences of discrimination at structural level could be understood by Foucault’s biopolitics and state racism, where biopolitics refers to the social control and power disseminated through social body, such as healthcare and is regarded as the norm. This gives rise to the state racism which becomes one of basic dimensions of social normalization,^50^ focusing on the superiority of dominant culture over the another who are culturally different from majority. Such type of structural racism that prevails in European health care, is normalized and is enacted through invisible, subtle practices by HPs (consciously or unconsciously) that leads to unequal access to treatment.^51^ This further leads to perceived racism, as stated by participants to be treated differently from the ethnic Norwegians and is associated with lack of trust in healthcare and refrain from seeking treatment.

Understanding barriers is critical to ensure lower drop-out rates and facilitate adequate use of treatment.^35^ The participants stated that receiving treatment from culturally competent HPs facilitated their treatment process, as their needs were understood during the counseling and healing process. This finding is consistent with previous studies^44,52^ and a review,^53^ where having HPs that were sensitive to cultural nuances was seen as more effective. This could be correlated with an approach of interculturalization of mental health services, which entails adopting treatment according to the patient’s cultural contexts and needs.^54^ Further, our participants had positive experiences with HPs of immigrant background, in line with a study by Salami et al.,^47^ especially if the HPs also had an understanding of Norwegian culture. In addition, HPs with war trauma experiences were considered as facilitators that motivated the participants to complete the treatment and gave them a sense of belonging.

Furthermore, the positive experience of being seen as a person and not as a diagnosis and of having one’s needs understood based on one’s culture, values and beliefs were appreciated by the participants and increased treatment engagement. This is similar to the approach of person-centeredness and individualization of SUD treatment services,^32,33^ which is now part of the national guidelines for SUD treatment.^34,55^ In a recent Norwegian study, participants’ narratives suggested that HPs who used “personal connection” and saw them as persons beyond their substance use problems were considered facilitators for treatment.^56^ In addition, participants acknowledged aftercare as an important factor to prevent relapses and improve treatment outcomes. Aftercare could be seen as long-term monitoring and support in SUD treatment^56^ and could be individualized to meet the needs of persons of immigrant background. Also, aftercare could be understood as follow-up care in treatment models which allows individuals to cope and regain a meaningful life when they are no longer in treatment settings, along with having a sense of being a contributing member of their community.^57^ This process can also aid in overcoming stigma by developing resilience toward stigma and/or actively fighting against it and can provide people with MHD/SUD with a sense of empowerment and control over their lives by exercising their rights and responsibilities as other citizens.^25^

Further, participants’ experiences revealed that the greater their awareness about the consequences of SUD and MHD, the higher was their engagement to treatment. It was also reported that many of their co-users were unaware that care was available and hence did not initiate treatment. Fong et al. suggested that creating alternative 12-step groups focusing primarily on support and education and less on confrontation would facilitate treatment,^45^ which is in line with participants’ experiences in our study. In addition, immigrants’ perceptions of the need for treatment were dependent on social embeddedness,^44^ hence strategies for reducing stigma within the social context may facilitate treatment among immigrants. Another treatment facilitator is the supportive network of peers and family.^48^ Being open with family and friends harnesses help-seeking enablers,^44^ along with raising the family’s mental health literacy,^47^ which is in agreement with the participants’ experiences. Further, having a supportive network gave the participants a feeling of inclusion.

Lastly, our study was able to include only men due to the challenges in recruiting immigrant women with co-occurring SUD and MHD. We had initially planned to recruit both women and men who met the inclusion criteria. We argue that the recruitment of immigrant men was extremely challenging due to the hidden nature and stigma attached to SUDs and/or MHDs. Our participants reported that there were many men and women with a similar diagnosis but they were not willing to participate in the study because of fear of being recognized and detected within their small communities in Norway. We believe that this fear and stigma are probably even more prevalent among immigrant women with co-occurring SUD and MHD, due to their perceived potential risk of greater harm if they are detected and identified and different cultural norms. In addition, due to experiences of shame, discrimination, and marginalization, immigrant women are less likely to report their SUD and/or MHD and are less likely to access the available care and treatment.^58^

## Limitations and strengths

This qualitative study provides insights into treatment experiences of immigrant men living with co-occurring SUD and MHD in Norway, which to our knowledge has not been previously explored. The results are based on our participants’ experiences and may be argued about the relevance beyond the local context. However, in exploring subjective experience involves focusing on the meaning of the participants, which may be transferred to other contexts and other people. Moreover, these insights are believed to be of relevance for future research. Further, we argue that our results provide insights into the experiences of a group of persons who are considered hard to reach and often stigmatized. Furthermore, the credibility in our study was enhanced by collaborating with a competency group in all stages of the study, starting from writing the protocol, preparing the study, recruiting the participants, analyzing the data, to compiling the results. In addition, in interview settings, both the participants and the first author were non-ethnic Norwegians, which facilitated the interview process, where the participants could trust the interviewer and feel connected, which helped to provide meaningful data. This could be understood as “diversity in proximity”, meaning that interaction between migrant researcher and migrant participant is effective when both of them can recognize the ties that bind and the social fissure that divides in a host country.^59^ We also believed that “diversity in proximity” enhanced the credibility of our study. Lastly, this study could only recruit immigrant men because of the challenges in recruiting immigrant women living with co-occurring SUD and MHD.

## Conclusion and future recommendations

Immigrant men living with co-occurring SUD and MHD interpreted their lived experiences of treatment in mental health and addiction services in Norway as both negative and positive. Lack of connection and individually tailored treatment along with stigma were important ongoing barriers to treatment and hence led to low treatment engagement. However, HPs with multicultural competence, aftercare and strategies for raising awareness and reducing stigma acted as facilitators to treatment, increasing treatment engagement. Improving the health of immigrants would benefit Norway and other countries, as migration is increasing worldwide. Hence, we argue that the insights from the participants are timely and that the knowledge from their treatment experiences can broaden the perspectives of practitioners and policy makers to provide more culturally tailored services. Further, we suggest that strategies that reduce barriers to treatment will require increased prevention and education efforts tailored to individual needs. We also suggest that treatment engagement may increase with a greater emphasis on strategies that provide more person-centered and culturally competent services. We suggest future research on how to better understand the impact of these barriers on the diagnosis of individuals and their participation in the society.

## Appendix A

**Table 1.** Description of participants.

Participant	Age in years	Region of origin	Age at migration to Norway	Age when starting to use substances	Drop-out from treatment
1	33	Middle East	1.5 y	12	Several times
2	42	West Africa	7 y	14	Twice
3	32	Middle East	5 mo	12	Several times
4	25	East Africa	16 y	17	Once
5	Around 30 (not confirmed)	African descent	12 y	17	Several times
6	38	South Asia	Born in Norway	12	Several times
7	29	South Asia	Born in Norway	19	Once
8	42	Middle East	11 y	15	Several times
9	53	Middle East	21 y	8	Several times
10	39	Middle East	24 y	16	Several times

## References

[1] Abebe DS Lien L Hjelde KH. What we know and don’t know about mental health problems among immigrants in Norway. J Immigr Minor Health. 2014;16:60-67. 23117694 10.1007/s10903-012-9745-9

[2] SSB. Immigrants and Norwegain-born to immigrant parents. Statistics Norway. Published 2020. Accessed March 9, 2020. https://www.ssb.no/en/befolkning/statistikker/innvbef.

[3] Chen W Hall BJ Ling L Renzaho AM. Pre-migration and post-migration factors associated with mental health in humanitarian migrants in Australia and the moderation effect of post-migration stressors: findings from the first wave data of the BNLA cohort study. Lancet Psychiatry. 2017;4:218-229. 28161455 10.1016/S2215-0366(17)30032-9

[4] Rossow I Bramness JG. The total sale of prescription drugs with an abuse potential predicts the number of excessive users: a national prescription database study. BMC Public Health. 2015;15:288. 25885781 10.1186/s12889-015-1615-7 PMC4377902

[5] Long EC Aggen SH Neale MC, et al The association between personality disorders with alcohol use and misuse: a population-based twin study. Drug Alcohol Depend. 2017;174:171-180. 28334662 10.1016/j.drugalcdep.2017.01.022 PMC5497569

[6] Torvik FA Rosenström TH Ystrom E, et al Stability and change in etiological factors for alcohol use disorder and major depression. J Abnorm Psychol. 2017;126:812-822. 28541064 10.1037/abn0000280 PMC5546937

[7] Carrà G Johnson S Crocamo C, et al Psychosocial functioning, quality of life and clinical correlates of comorbid alcohol and drug dependence syndromes in people with schizophrenia across Europe. Psychiatry Res. 2016;239:301-307. 27046394 10.1016/j.psychres.2016.03.038

[8] Davidson L Andres-Hyman R Bedregal L Tondora J Frey J Kirk TA Jr. From “double trouble” to “dual recovery”: integrating models of recovery in addiction and mental health. J Dual Diagn. 2008;4:273-290.

[9] NIPH. Substance use disorders in Norway. Norwegian Institute of Public Health. Published August 8, 2016. Updated December 9, 2019. Accessed April 12, 2020. https://www.fhi.no/en/op/hin/mental-health/intoxicants-and-substance-use/

[10] WHO. Mental disorders. World Health Organization. Published November 28, 2019. Accessed January 11, 2020. https://www.who.int/en/news-room/fact-sheets/detail/mental-disorders

[11] Mladovsky P Rechel B Ingleby D McKee M. Responding to diversity: an exploratory study of migrant health policies in Europe. Health Policy. 2012;105:1-9. 22306024 10.1016/j.healthpol.2012.01.007

[12] Harris S Dykxhoorn J Hollander A-C Dalman C Kirkbride JB. Substance use disorders in refugee and migrant groups in Sweden: a nationwide cohort study of 1.2 million people. PLoS Med. 2019;16:e1002944. 31689291 10.1371/journal.pmed.1002944 PMC6830745

[13] Lundgren L Brännström J Armelius B-Å Chassler D Morén S Trocchio S. Association between immigrant status and history of compulsory treatment in a national sample of individuals assessed for drug use disorders through the Swedish public welfare system. Subst Use Misuse. 2012;47:67-77. 22122072 10.3109/10826084.2011.628736

[14] Mladovsky P. Migrant health in the EU. Eurohealth (Lond). 2007;13:9.

[15] Sandøy TA. Group solidarity in a hostile milieu: immigrant experiences in a street-based drug scene. Drugs (Abingdon Engl). 2015;22:232-238.

[16] Abebe DS Lien L Elstad JI. Immigrants’ utilization of specialist mental healthcare according to age, country of origin, and migration history: a nation-wide register study in Norway. Soc Psychiatry Psychiatr Epidemiol. 2017;52:679-687. 28378064 10.1007/s00127-017-1381-1

[17] Ivert A-K Merlo J Svensson R Levander MT. How are immigrant background and gender associated with the utilisation of psychiatric care among adolescents? Soc Psychiatry Psychiatr Epidemiol. 2013;48:693-699. 23001409 10.1007/s00127-012-0589-3

[18] Mulder CL Koopmans GT Selten J-P. Emergency psychiatry, compulsory admissions and clinical presentation among immigrants to the Netherlands. Br J Psychiatry. 2006;188:386-391. 16582067 10.1192/bjp.188.4.386

[19] Brorson HH Arnevik EA Rand-Hendriksen K Duckert F. Drop-out from addiction treatment: a systematic review of risk factors. Clin Psychol Rev. 2013;33:1010-1024. 24029221 10.1016/j.cpr.2013.07.007

[20] Dixon LB Holoshitz Y Nossel I. Treatment engagement of individuals experiencing mental illness: review and update. World Psychiatry. 2016;15:13-20. 26833597 10.1002/wps.20306 PMC4780300

[21] Vandevelde S Vanderplasschen W Broekaert E. Cultural responsiveness in substance-abuse treatment: a qualitative study using professionals’ and clients’ perspectives. Int J Soc Welf. 2003;12:221-228.

[22] Verdurmen JE Smit F Toet J Van Driel HF Van Ameijden EJ. Under-utilisation of addiction treatment services by heroin users from ethnic minorities: results from a cohort study over four years. Addict Res Theory. 2004;12:285-298.

[23] De Kock C Decorte T Vanderplasschen W Derluyn I Sacco M Studying ethnicity, problem substance use and treatment: from epidemiology to social change. Drugs (Abingdon Engl). 2017;24:230-239.

[24] Miller WR Rollnick S. Motivational Interviewing: Helping People Change. Guilford Press; 2012.

[25] Davidson L Tondora J O’Connell MJ Kirk T Jr Rockholz P Evans AC. Creating a recovery-oriented system of behavioral health care: moving from concept to reality. Psychiatr Rehabil J. 2007;31:23-31. 17694712 10.2975/31.1.2007.23.31

[26] McCormack B McCance T. Person-Centred Nursing: Theory And Practice. John Wiley & Sons; 2011.

[27] McWhinney IR ‘An acquaintance with particulars. . .’. Fam Med. 1989;21:296-298. 2753257

[28] Cassell EJ. The person in medicine. Int J Integr Care. 2010;10:5 10.5334/ijic.489 PMC2834910 20228916

[29] Mjølstad BP Kirkengen AL Getz L Hetlevik I. What do GPs actually know about their patients as persons? Eur J Pers Cent Healthc. 2013;1:149.

[30] McCormack B van Dulmen S Eide H Skovdahl K Eide T. Person-Centred Healthcare Research. John Wiley & Sons; 2017.

[31] Kour P Lien L Kumar B Biong S Pettersen H. Coping and negotiating a sense of self: immigrant men’s experiences of living with co-occurring substance use and mental health disorders in Norway. Am J Psychiatr Rehabil. 2020;22:43-63.

[32] Friedmann PD Hendrickson JC Gerstein DR Zhang Z. The effect of matching comprehensive services to patients’ needs on drug use improvement in addiction treatment. Addiction. 2004;99:962-972. 15265093 10.1111/j.1360-0443.2004.00772.x PMC2761625

[33] Hser Y-I Polinsky ML Maglione M Anglin MD. Matching clients’ needs with drug treatment services. J Subst Abuse Treat. 1999;16:299-305. 10349602 10.1016/s0740-5472(98)00037-3

[34] NDH. National Guidelines for Treatment and Rehabilitation of Substance use Disorders. Norwegian Directorate of Health (NDH); 2017.

[35] Falgas-Bague I Wang Y Banerjee S, et al Predictors of adherence to treatment in behavioral health therapy for Latino immigrants: the importance of trust. Front Psychiatry. 2019;10:817. 31780971 10.3389/fpsyt.2019.00817 PMC6856783

[36] Pinedo M Zemore S Rogers S. Understanding barriers to specialty substance abuse treatment among Latinos. J Subst Abuse Treat. 2018;94:1-8. 30243409 10.1016/j.jsat.2018.08.004 PMC6157272

[37] Trivedi P Wykes T. From passive subjects to equal partners: qualitative review of user involvement in research. Br J Psychiatry. 2002;181:468-472. 12456515 10.1192/bjp.181.6.468

[38] Faulkner A. The Ethics of Survivor Research: Guidelines for the Ethical Conduct of Research Carried Out by Mental Health Service Users and Survivors. Policy Press; 2004.

[39] Beresford P. User involvement in research: exploring the challenges. NT Res. 2003;8:36-46.

[40] Patton MQ. Qualitative Research & Evaluation Methods: Integrating Theory and Practice. Sage Publications; 2014.

[41] Kvale S Brinkmann S. Interviews: Learning the Craft of Qualitative Research Interviewing. Sage Publications; 2009.

[42] Malterud K Siersma VD Guassora AD. Sample size in qualitative interview studies: guided by information power. Qual Health Res. 2016;26:1753-1760. 26613970 10.1177/1049732315617444

[43] Malterud K. Systematic text condensation: a strategy for qualitative analysis. Scand J Public Health. 2012;40:795-805. 23221918 10.1177/1403494812465030

[44] McCann TV Mugavin J Renzaho A Lubman DI. Sub-Saharan African migrant youths’ help-seeking barriers and facilitators for mental health and substance use problems: a qualitative study. BMC Psychiatry. 2016;16:275. 27484391 10.1186/s12888-016-0984-5 PMC4971683

[45] Fong TW Tsuang J. Asian-Americans, addictions, and barriers to treatment. Psychiatry (Edgmont). 2007;4:51-59. PMC2860518 20428303

[46] Sandhu S Bjerre NV Dauvrin M, et al Experiences with treating immigrants: a qualitative study in mental health services across 16 European countries. Soc Psychiatry Psychiatr Epidemiol. 2013;48:105-116. 22714866 10.1007/s00127-012-0528-3

[47] Salami B Salma J Hegadoren K. Access and utilization of mental health services for immigrants and refugees: perspectives of immigrant service providers. Int J Ment Health Nurs. 2019;28:152-161. 29984880 10.1111/inm.12512

[48] Derr AS. Mental health service use among immigrants in the United States: a systematic review. Psychiatr Serv. 2016;67:265-274. 26695493 10.1176/appi.ps.201500004 PMC5122453

[49] Gee GC Delva J Takeuchi DT. Relationships between self-reported unfair treatment and prescription medication use, illicit drug use, and alcohol dependence among Filipino Americans. Am J Public Health. 2007;97:933-940. 16809581 10.2105/AJPH.2005.075739 PMC1854877

[50] Foucault M Discipline and Punish: The Birth of the Prison (Trans. Alan Sheridan). Vintage; 1977: 227.

[51] Hamed S Thapar-Björkert S Bradby H Ahlberg BM. Racism in European health care: structural violence and beyond. Qual Health Res. 2020;30:1662-1673. 32546076 10.1177/1049732320931430 PMC7410275

[52] Masson CL Shopshire MS Sen S, et al Possible barriers to enrollment in substance abuse treatment among a diverse sample of Asian Americans and Pacific Islanders: opinions of treatment clients. J Subst Abuse Treat. 2013;44:309-315. 22985677 10.1016/j.jsat.2012.08.005 PMC3545039

[53] Abebe DS. Public health challenges of immigrants in Norway: a research review. NAKMI Report. 2010;2:41-48.

[54] De Jong JT Van Ommeren M. Mental health services in a multicultural society: interculturalization and its quality surveillance. Transcult Psychiatry. 2005;42:437-456. 16268237 10.1177/1363461505055625

[55] NIDA. Principles of Drug Addiction Treatment: A Research-Based Guide. 3rd ed. National Institute on Drug Abuse; 2018.

[56] Pettersen H Landheim A Skeie I, et al Helpful ingredients in the treatment of long-term substance use disorders: a collaborative narrative study. Subst Abuse. 2019;13:1178221819844996. 10.1177/1178221819844996 PMC6487766 31065215

[57] Davidson L O’Connell M Tondora J Styron T Kangas K. The top ten concerns about recovery encountered in mental health system transformation. Psychiatr Serv. 2006;57:640-645. 16675756 10.1176/ps.2006.57.5.640

[58] Gueta K. A qualitative study of barriers and facilitators in treating drug use among Israeli mothers: an intersectional perspective. Soc Sci Med. 2017;187:155-163. 28689089 10.1016/j.socscimed.2017.06.031

[59] Ganga D Scott S Cultural “insiders” and the issue of positionality in qualitative migration research: moving “across” and moving “along” researcher-participant divides. In Forum Qualitative Sozialforschung/Forum: Qualitative Social Research; 2006;7:3.

